# Postural stability reveals cognitive load of holding an intention in mind

**DOI:** 10.1038/s41598-025-26207-6

**Published:** 2025-11-26

**Authors:** Maximilian Haas, Christian Chicherio, Matthias Kliegel, Delphine Fagot

**Affiliations:** 1 Faculty of Psychology and Educational Sciences, University of Geneva, Geneva, Switzerland; 2 Centre for the Interdisciplinary Study of Gerontology and Vulnerability, University of Geneva, Geneva, Switzerland; 3 UniDistance Suisse, Brig, Switzerland; 4 Hôpitaux Universitaires de Genève, Geneva, Switzerland

**Keywords:** Prospective memory, Cognitive load, Balance, Aging, Dual-task, Center of pressure, Neuroscience, Psychology, Risk factors

## Abstract

It is well known that walking while talking may be challenging, but does merely keeping a future intention in mind (i.e., prospective memory, PM), even without execution, impose cognitive costs on postural stability? Reaction times and accuracy in a primary cognitive activity used so far may not fully capture these hidden costs, whereas center of pressure (COP) fluctuations offer a promising task-external indicator of cognitive load due to shared cognitive-postural resources. Fifty-three younger (18–30 years) and 47 older adults (60–86 years) performed an arrow task while standing on a force platform. Cognitive load was manipulated across two task-load conditions (low vs. high) and three PM conditions: (1) no PM instruction, (2) an instructed but unexecuted PM task (intended-PM), and (3) an instructed and executed PM task (executed-PM). The Cognition-Balance-Cost-Index (CBCI), integrating reaction times and COP, quantified cognitive-postural resource allocation. Results showed that younger adults’ CBCI remained stable, with only a moderate increase in executed-PM under high load. In contrast, older adults exhibited significantly higher CBCI scores across all conditions, peaking in the executed-PM condition. Notably, intended-PM under high load reduced CBCI in older adults, suggesting compensatory resource allocation, and highlighting postural stability as a valuable, non-invasive indicator of cognitive load.

## Introduction

Walking while talking is known to be difficult, particularly in non-routine situations and in case of cognitive highly demanding conversations^1–3^. The effect of dual-task distraction on gait is particularly relevant regarding fall risk in aging^4,5^ or in populations with reduced motor control and/or cognitive resources such as patients suffering brain trauma or those with Alzheimer’s or Parkinson’s disease^6–9^. Although actively engaging in concurrent tasks has been shown to affect gait and postural control, it currently remains unknown whether merely keeping an intention in mind for future execution, even without eventually accomplishing it, does compete with resources allocated to postural stability.

Prospective memory (PM) describes the ability to remember performing an intention at a future point in time, such as remembering to attend an appointment or to take medication with a meal^10,11^. More than half of all daily memory failures are related to PM, underscoring its prevalence in everyday life and its crucial role for functional independence especially in older age^12,13^. PM tasks generally are divided into two categories, namely *event-based* tasks, which are triggered by an external cue (e.g., remembering to buy bread when passing by a bakery), and *time-based* tasks, where the intention relies on internal or time-based cues (e.g., remembering to call my mother on her birthday). This division is closely related to the underlying processes of intention retrieval (i.e., bottom-up retrieval for event-based and top-down processing in time-based PM).

### Cognitive load, attentional resources, and PM performance

Conceptually, it has been argued that successful PM performance may require different degrees of cognitive resources, depending on whether top-down or bottom-up processes are involved in prospective remembering. According to the Multiprocess Theory^14,15^, successful PM performance is dependent on both top-down monitoring processes and spontaneous bottom-up retrieval, allowing individuals to complete a PM task with varying degrees of cognitive effort. In contrast, the Preparatory Attentional and Memory (PAM) process theory has emphasized the need for dedicated attentional resources to continuously monitor for PM cues, suggesting that PM performance necessarily relies on top-down resource allocation, thus increasing cognitive load^16,17^. Although evidence in favor of bottom-up spontaneous retrieval and execution pathways exists, it is argued that maintaining PM intentions, especially without achieving them, places a continuous demand on cognitive resources^18,19^. As a consequence, the cognitive load associated with maintaining the PM intention may spill over on concurrent tasks, such as through slowing reaction times or reducing accuracy, thus imposing a cost in the ongoing activity and particularly so in older adults, who may experience declines in attentional and executive resources with age^20–23^.

A recent study^24^ explored how cognitive load and task focality jointly influence PM performance. Using fMRI, the researchers analyzed brain activity while participants engaged in tasks with varying load (i.e., monitoring one or two PM targets) and focality (i.e., focal or non-focal). Results showed that high-load conditions activated the left intraparietal sulcus, supporting the hypothesis that cognitive load impacts brain regions associated with attentional control. Under low-load conditions, non-focal tasks triggered activity in the ventral occipito-temporal cortex, suggesting that different neural pathways are engaged based on task demands. Behaviorally, participants experienced slower reaction times with increased load, particularly in focal tasks, implying that cognitive resources may shift from automatic to controlled monitoring as load increases.

Although commonly used ongoing task-based indicators of cognitive load (i.e., reaction time and accuracy) are valuable, they may not fully capture the underlying cognitive effort, such as more subtle, task-external cognitive costs which manifest outside direct performance-related measures.

### Postural stability and cognitive load

Postural stability, and specifically ‘Center of Pressure’ (COP), has recently gained attention in this regard as it appears to share common resources with cognitive functioning^25–27^. COP represents the point at which the total force acting on a person’s feet is concentrated, and is therefore considered fundamental to human movement and balance. COP measurements typically examine sway in two primary directions, namely the medio-lateral axis (i.e., side-to-side; ML) and the anterior-posterior axis (i.e., front-to-back; AP). Aging is associated with an increase in sway, particularly along the AP axis, due to declines in sensory processing and coordination of muscle responses, factors which reduce the speed and efficiency of postural adjustments in older age^28–32^. Although the ML axis is less impacted by aging, increased ML sway has been associated with a higher risk of falls in older adults^33,34^.

Fluctuations in COP (i.e., increased sway) in response to cognitively demanding tasks could suggest that postural control competes with mental resources, providing an indirect, non-invasive marker of cognitive load. In fact, studies using dual-task paradigms have demonstrated that cognitive and postural demands can draw on shared resources, sometimes resulting in impaired cognitive performance when more resources are directed toward postural control. In one study, the researchers asked participants to complete a cognitive task while either receiving or not receiving vibratory stimulation to their lower legs^35^. Results indicated that cognitive performance declined when additional resources were to be allocated to postural stability. Research exploring the opposite direction (i.e., the influence of cognitive load on stability) has provided mixed findings, with some studies showing increased sway under cognitive load, others showing improved stability, and still others reporting no effect^36–39^. This inconsistency may partly relate to age-related differences, as older adults, who often show greater sway and instability than younger adults, tend to prioritize postural control over cognitive performance, particularly when the cognitive tasks are demanding^40–46^. In fact, it has been shown that older adults display lower performance in dual-task paradigms due to mechanisms related to a decline with age of divided attention in concurrent tasks^47–49^.

In sum, these findings highlight COP as a valuable potential indicator for understanding task-external costs of cognitive load. Moreover, no study to date has investigated the link between postural stability and costs in PM. Going beyond common task-related measures of reaction times and accuracy, examining COP as an indicator of postural stability during PM tasks may offer a promising approach to detect hidden costs of holding and maintaining PM intentions, particularly in older adults. While reaction time and COP have mostly been examined independently in dual-task paradigms^50,51^, a few exceptions have begun to explore composite indices. For example, a ‘gait and cognition pooled index’ combining gait velocity and cognitive scores was used to enhance sensitivity in pre-dementia detection^52^. However, to our knowledge, no prior study has integrated reaction time and COP into a unified measure specifically targeting load-detection associated with PM intention maintenance.

### Research questions and study goals

The present study aimed to examine whether COP may serve as a task-external indicator to detect cognitive load induced by maintaining (deactivated) PM intentions. Specifically, we investigated whether the postural imbalance associated with maintaining a PM intention mirror (or complement) established task-based costs, such as increased reaction times and decreased accuracy, particularly when attentional resources are limited. Additionally, we sought to determine whether a commercially available force platform could reliably detect changes in postural stability due to variations in PM load, and whether these changes in postural imbalance are influenced by task difficulty. Given the lack of an established framework that quantitatively integrates cognitive performance and postural stability, we introduce the Cognition-Balance Cost Index (CBCI) as a proof-of-concept measure that combines reaction times and COP into a single, dimensionless index. CBCI was designed to capture the proportional trade-off between costs in cognitive versus postural domains, thereby providing an integrated perspective on cognitive-motor resource allocation with increasing task load.

## Methods

### Participants

Fifty-three younger adults (*M* = 23.15, *SD* = 2.97; age range: 18–30 years, 29 women) and 47 older adults (*M* = 69.53, *SD* = 6.18; age range: 60–86 years, 30 women) participated in the present study. All participants (a) were aged 18 or above; (b) were native French speakers or had very good written and oral knowledge of French language with at least five years of daily practice, (c) presented (corrected to) normal vision, and (d) gave written informed consent to participate in this study, which was approved by the ethics committee of the Faculty of Psychology and Educational Sciences of the University of Geneva and conducted in accordance with the principles of the Declaration of Helsinki. Participation was voluntary; younger adults were eligible to receive course credits in exchange for their participation if they were enrolled as undergraduate students in Psychology at the University of Geneva. Table 1 shows participants’ socio-demographic data and their performances on the (cognitive) background measures described below.

**Table 1:** Participants’ sociodemographic information and performances on (cognitive) background measures.

	Younger adults (*N* = 53)	Older adults (*N* = 47)
Age (years)***	23.15 (2.97)	69.53 (6.18)
Gender (women/men)	29 / 24	30 / 17
Education (years)	15.84 (2.43)	16.07 (4.72)
MillHill vocabulary	33.28 (4.07)	34.68 (3.37)
Raven matrices (reasoning)***	25.17 (2.95)	20.38 (3.74)
Depression**	5.77 (2.48)	3.96 (2.63)
PAS	4.50 (2.10)	4.19 (2.15)
HLAQ (MET-h/week)***	23.49 (25.50)	63.08 (52.39)

Results are reported as means with their respective (standard deviation).

*Depression* Score on the Geriatric Depression Scale (covariate), *PAS* Current level of physical activity measures as equivalent of hours per week spent on regular weekly physical practice as assessed with the NASA/JSC Physical Activity Scale (covariate); *HLAQ* Mean level of physical activity expressed as average energy expenditure in physical and leisure activities (in MET-hours/week) as assessed with the Historical Leisure Activity Questionnaire (covariate).

*p < .05. **p < .01. ***p < .001.

### Material and general procedure

After signing the informed consent, all participants were administered the cognitive task in an individual assessment session lasting two hours. The cognitive task was presented on a computer using E-Prime^®^; see section *Cognitive Task* below for a detailed description of the manipulation and the task conditions. While performing the cognitive task, the participant was standing on a force platform (Wii Balance Board RVL-021, Bigben interactive Balance board for Wii)^53^ connected via Bluetooth to the computer. The force platform allowed for a fine-grained assessment of movement and changes in body position during task performance. As the measure of postural stability is likely to be influenced by participants’ level of physical activity, all participants were asked to rate both their recent physical activity level over the last six months using the NASA/JSC Physical Activity Scale^54^, as well as their level of average energy expenditure across the lifespan using the Historical Leisure Activity Questionnaire^55^. Additionally, we assessed participants’ height and body weight to compute their individual body mass index (BMI). All three measures of physical fitness served as covariates for the statistical analyses.

### Cognitive task paradigm

Participants’ cognitive performance was assessed using an arrow task paradigm, in which a strongly activated dominant response must be suppressed to provide the correct answer^56,57^. In the present study, the paradigm comprised right (→) and left (←) pointing black arrows presented in one of two locations, namely left or right along the medial-horizontal axis of a computer screen. Participants were asked to always respond according to the direction indicated by the arrow, independently of its spatial location. In detail, this gave rise to two distinct types of stimuli, as defined by the relationship between their location and direction of each arrow (i.e., ‘congruent stimuli’ when both the location and the pointed direction were identical, and ‘incongruent stimuli’ when the location of the arrow and its pointed direction were opposite). All stimuli were presented on a grey background screen and were equally distributed across location and direction.

The paradigm was split in blocks of 120 trials according to two task instructions, namely (1) a part in which only congruent stimuli were presented and the participants were asked to detect the direction pointed by the arrow (i.e., ‘Detection task’), and (2) a part in which both congruent (⅔, *n* = 80) and incongruent (⅓, *n* = 40) stimuli were presented and participants had to respond according to the direction pointed by the arrow while ignoring its spatial location (i.e., ‘Inhibition task’).

### PM task manipulation

Additionally, we manipulated the cognitive complexity in each of the tasks: In a first condition, participants were only required to indicate the direction to which the arrow pointed, independently of its spatial location (i.e., ongoing-task only; ‘Simple condition’).

At the second level of cognitive complexity, participants were also required to indicate the direction to which the arrow pointed (i.e., they continued performing the ongoing task), but an additional PM task instruction was provided: In fact, participants were instructed to simultaneously press the right and left keys in case the color of the arrow was red. Yet, in this condition, this event was instructed but never appeared (i.e., Intended prospective memory condition; I-PM).

The third level is similar to the second, with the exception that the event actually occurred in 10% of all trials (i.e., Executed prospective memory condition; E-PM).

**Fig. 1:**
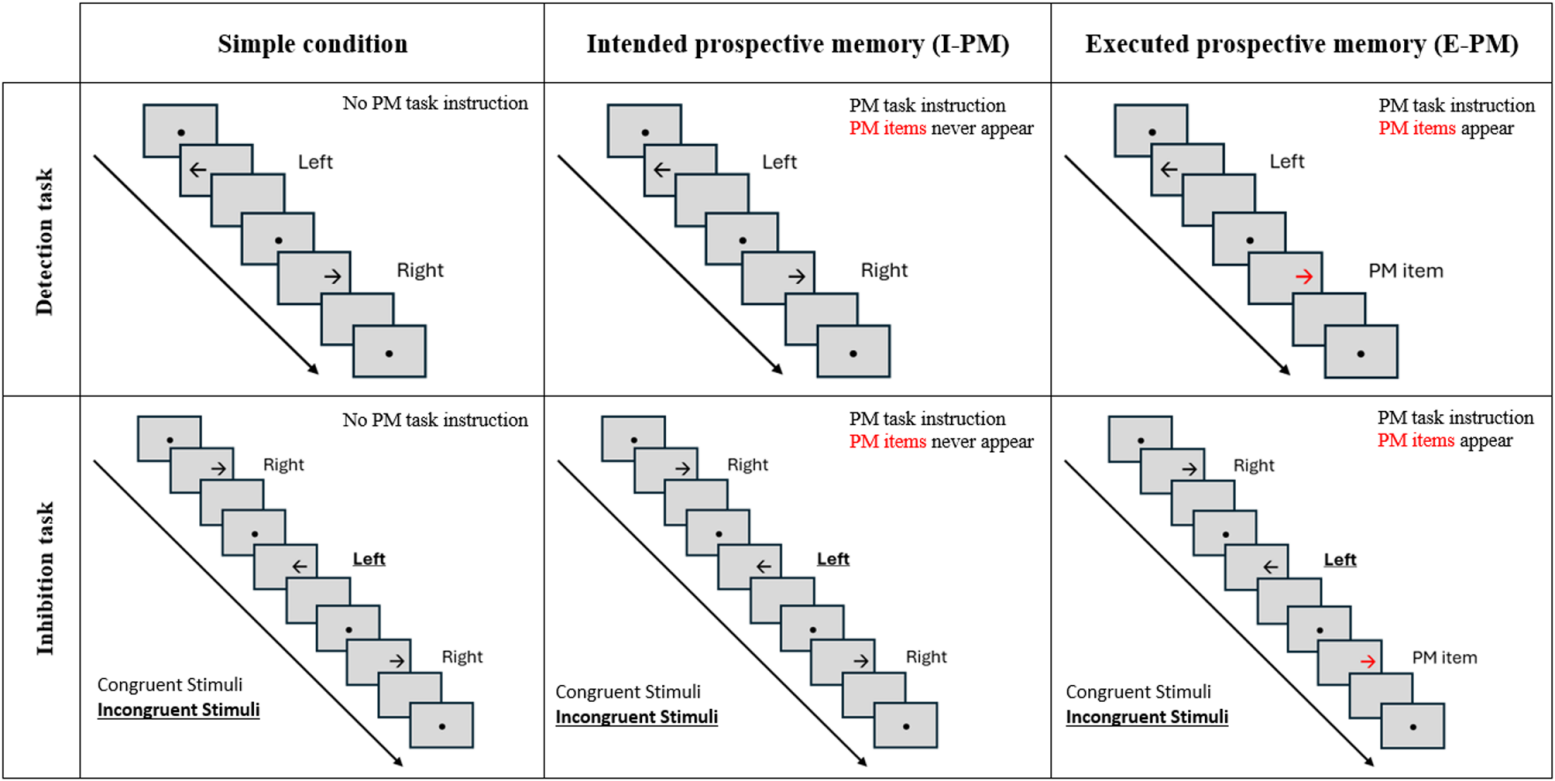
Illustration of the task load and cognitive complexity conditions of the cognitive paradigm. Congruent stimuli = location and pointed direction are identical (i.e., arrows pointing to the left are always shown on the left side of the screen); Incongruent stimuli = location and pointed direction are opposite (i.e., arrows pointing to the left are shown on the right side of the screen); PM items = arrows colored in red trigger secondary task behavior. Cognitive complexity increases from left to right (simple < I-PM < E-PM); task load increases from upper to lower row (Detection task < Inhibition task). Sequence for each trial: a black fixation point appears at the center of the screen (mean duration: ± 500 ms), then the stimulus appeared and remained on the screen until the onset of the participant’s response, then blank screen (1000 ms) following the onset of the participants’ response or after a maximum of 2500 ms.

In total, participants thus completed 6 blocks of 120 trials each, namely (1) Detection task – Simple condition; (2) Detection task – I-PM condition; (3) Detection task – E-PM condition; (4) Inhibition task – Simple condition; (5) Inhibition task – I-PM condition, and (6) Inhibition task – E-PM condition (see Fig. 1). The order of the trials within each task and condition was pseudo-randomized for all participants and the order of the blocks was counterbalanced across individuals. The task started with a practice of ten trials, with stimuli and timing identical to those of the experimental blocks. For each trial, a black fixation point appeared at the center of the screen for a mean duration of 500 ms, varying randomly between 300 and 700 ms (in steps of 100 ms). The stimulus appeared and remained on the screen until the onset of the participant’s response. Afterwards, the screen went blank for 1000 ms following the onset of the participants’ response or after a maximum of 2500 ms and before proceeding to the next trial.

### Centre of pressure (COP)

In the present study, postural stability was measured by analyzing the Center of Pressure (COP) through a structured assessment protocol. Prior to data collection, key participant information (e.g., weight and height) and specific recording conditions (e.g., eyes open vs. eyes closed) were specified, along with details on the recording duration and sampling frequency. Each participant completed three COP measurement trials for each task condition, with recording times fixed at 300 s, resulting in 10,000 continuous data points per condition for each participant. Data were analyzed using the software KiKiBio^58^.

**Fig. 2:**
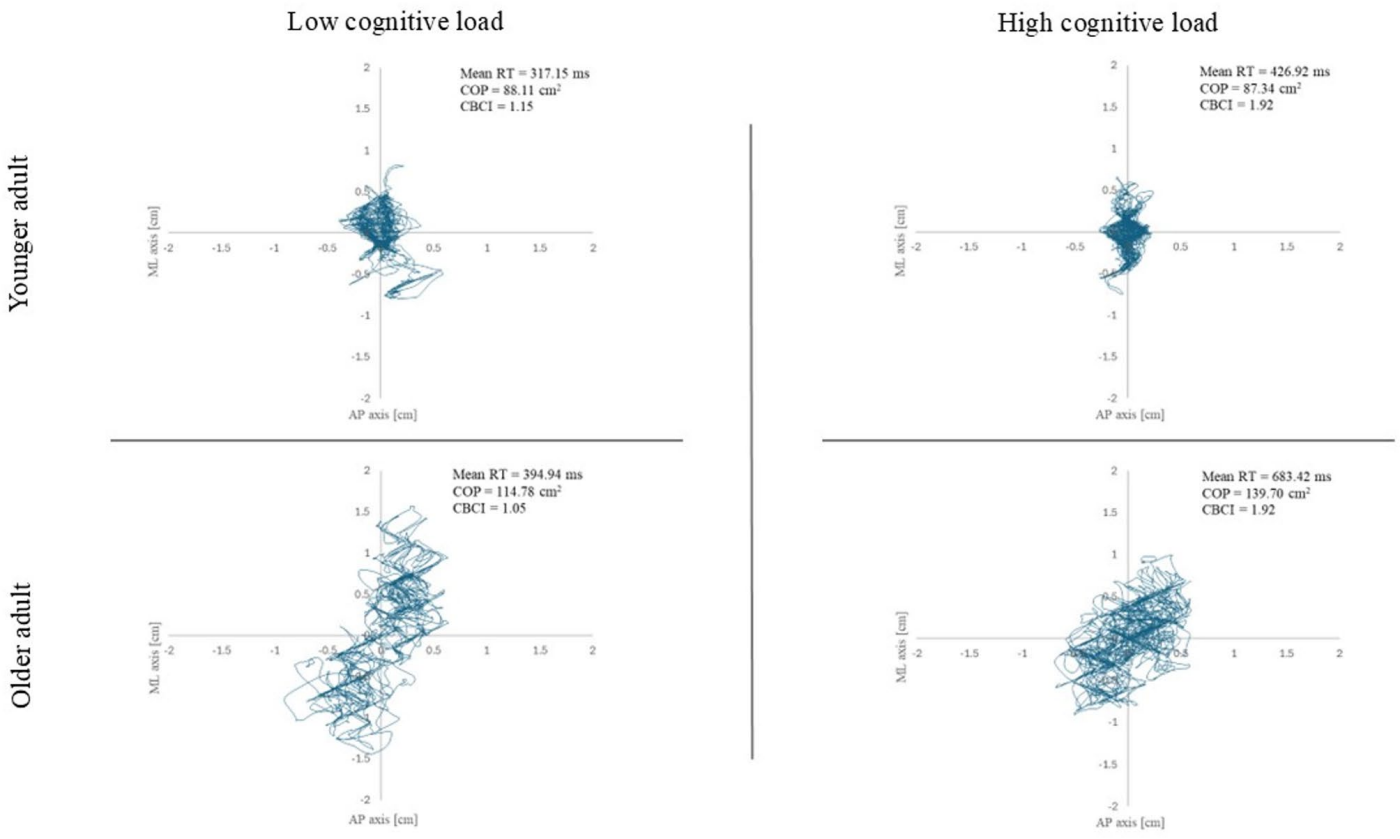
Illustration of the influence of cognitive load on the relationship between reaction times, centre of pressure, and the cognition-balance cost index in younger and older adults. AP axis = Anterior-Posterior movements (i.e., front-to-back); ML axis = Medio-Lateral movements (i.e., side-to-side); Mean RT = mean reaction time; COP = center of pressure; CBCI = Cognition-Balance Cost Index. At the example of data from one younger adult (upper row) and one older adult (lower row), the present figure illustrates changes in COP from the lowest cognitive load (i.e., Detection task—Simple condition) to the highest cognitive load combination (i.e., Inhibition task—E-PM condition). A similar increase in CBCI values can be explained by stronger preference for postural stability in both the younger and the older participant (i.e., pattern of movements on AP and ML axis has higher density in the high-load condition on the right).

The COP measurements were computed based on postural fluctuations along the anterior-posterior (AP) and medio-lateral (ML) axes (for an illustration in younger and older adults, see Fig. 2). The key COP metric included for the present analyses was the total COP displacement, defined by the cumulative distance covered by the participant’s COP along the AP and ML axis, expressed in centimeters.

To evaluate the impact of cognitive complexity on participants’ postural stability, a baseline measure of the COP was recorded prior to the cognitive task. Specifically, participants were instructed to stand as still as possible on a force platform twice for one minute each, with their eyes open, holding response triggers, and focusing on the computer screen without engaging in any additional tasks. The average of these two eyes-open measurements served as the baseline COP (E_0_), which provided a reference point for identifying any balance variations induced by the subsequent cognitive task conditions.

### Background measures

We administered the French version of the MillHill vocabulary scale^59^ and the progressive matrices test^60^ as a measure of participants’ global cognitive functioning. Furthermore, we used the Geriatric Depression Scale^61^ as a control of individuals’ current state of clinically relevant depressive symptoms, as well as NASA/JSC Physical Activity Scale (PAS)^54^ for participants’ current physical activity level and the Historical Leisure Activity Questionnaire (HLAQ)^55^ as measure of participants’ lifetime physical activity expenditure.

### Statistical analyses

Only correct response latencies were analyzed and RTs below 150 ms were excluded from the analyses. To determine if the COP measure can serve as a valid indicator of cognitive load in PM, we propose the Cognition-Balance Cost Index (CBCI) as a proof-of-concept measure, which reflects the trade-off between reaction time and postural imbalance:

$$\:CBCI=\frac{{\text{R}\tilde{\text{T}}}_{i\text{c}\text{t}}}{\text{C}\tilde{\text{O}}{\text{P}}_{\text{i}\text{ct}}}-1$$

with baseline-normalized values of reaction times $\:{\text{R}\tilde{\text{T}}}_{i\text{c}\text{t}}=\frac{{\text{R}\text{T}}_{ict}}{{\text{R}\text{T}}_{0}}$ and postural stability $\:\text{C} \tilde{\text{O}}{\text{P}}_{\text{i}\text{ct}}\text{=}\frac{{\text{C}\text{O}\text{P}}_{ict}}{{\text{C}\text{O}\text{P}}_{0}}$ for each individual *i* in the different tasks *t* (i.e., Detection vs. Inhibition) and complexity conditions *c* (i.e., Simple vs. I-PM vs. E-PM) relative to the mean performance of the younger adults in the baseline condition (Simple; low-load condition; subscript 0).

In detail, for each participant, condition (Simple vs. I-PM vs. E-PM), and task type (Detection vs. Inhibition), mean reaction times (RT_*i*ct_) and mean centre of pressure (COP_*i*ct_) path length were first normalized relative to the baseline condition performance (Simple, low-load condition in younger adults; subscript 0). The CBCI has then been defined as the ratio between normalized RT ($\:\text{R}\tilde{\text{T}}$) and normalized COP ($\:\text{C}\tilde{\text{O}}\text{P}$). By subtracting 1 from this ratio, the measure is centered at zero when weighted changes in RT and COP are comparable. This index thus allows to disentangle whether performance costs are more evident in reaction times (i.e., positive scores above 0) or in postural imbalance (i.e., negative scores below 0). Moreover, it offers the possibility to compare fluctuations between RT and COP across different conditions (e.g., a higher index standing for either higher mean RT or a lower COP). This formulation was chosen to reflect the proportional weighting of cognitive versus postural resources, acknowledging that dual-task costs may emerge in either or both domains. By adapting a baseline-normalized approach, this way of computing the CBCI isolates task-induced changes in resource allocation without conflating absolute group differences (e.g., older adults always slower, always more sway) with task-induced trade-offs – thus making cross-age comparisons fairer. As a ratio-based score of standardized values, the CBCI is a dimensionless indicator.

We conducted a repeated-measures ANCOVA with level of cognitive complexity (Simple vs. I-PM vs. E-PM) and task type (Detection task vs. Inhibition task) as within-subject factors, and age group (younger vs. older adults) as a between-subject factor. Covariates included (a) the presentation order of blocks, (b) the baseline COP in the eyes-open condition (E_0_), (c) the level of depressive symptoms, (d) individuals’ BMI, and (e) their levels of recent (PAS) and lifetime physical activity (HLAQ). In addition to the task-related covariates (a and b), depressive symptoms were considered due to their impact on cognitive functioning, whereas between-person differences in BMI and physical activity measures are known predictors of postural stability and motor performance. Together, these covariates control for individual differences in cognitive and motor capacity that could otherwise confound the observed effects of task load and age on CBCI. Hence, their inclusion ensured that age effects on CBCI can be attributed to induction of cognitive load and were not methodological artefacts. Bonferroni corrections were applied to all post-hoc analyses to adjust for multiple comparisons. Descriptive statistics for all performance indicators are provided in Table 2.

**Table 2:** Performance indicators for younger and older adults as function of task and cognitive complexity.

		Younger adults (*N* = 53)			Older adults (*N* = 47)		
		Simple	I-PM	E-PM	Simple	I-PM	E-PM
Accuracy	Detection task	0.99 (0.02)	0.99 (0.02)	0.97 (0.02)	1.00 (0.01)	1.00 (0.01)	0.99 (0.02)
	Inhibition task	0.90 (0.03)	0.91 (0.03)	0.93 (0.02)	0.92 (0.04)	0.91 (0.05)	0.92 (0.07)
Reaction times	Detection task	287.66 (33.28)	293.93 (35.99)	346.16 (43.93)	386.61 (70.05)	415.71 (80.48)	519.26 (120.17)
	Inhibition task	400.74 (50.47)	404.69 (58.63)	451.30 (70.78)	634.39 (129.90)	637.99 (139.66)	762.62 (192.81)
COP	Detection task	171.71 (143.73)	184.38 (205.12)	172.39 (87.05)	191.13 (100.20)	177.42 (72.33)	227.52 (198.82)
	Inhibition task	177.47 (116.43)	185.92 (121.68)	195.83 (271.47)	204.55 (120.56)	219.19 (114.31)	204.13 (156.24)
CBCI	Detection task	0.30 (0.53)	0.26 (0.54)	0.41 (0.55)	0.70 (0.70)	0.65 (0.74)	1.03 (0.88)
	Inhibition task	0.45 (0.55)	0.59 (0.59)	0.79 (0.80)	1.25 (0.97)	1.06 (0.82)	1.95 (1.41)

*Accurary* Proportion of correctly answered trials in the ongoing task, *Reaction Times* Reaction times in ms, *COP* Centre of pressure in cm^2^; *CBCI* Cognition-Balance Cost Index, *I-PM* Intended prospective memory condition (i.e., PM task is instructed but never performed; deactivated intentions), *E-PM* Executed prospective memory (i.e., PM task is instructed and has to be performed upon appearance of red arrows).

## Results

To confirm that any observed COP effects were attributable to the experimental manipulations, we conducted a first ANCOVA examining cognitive complexity (Simple vs. I-PM vs. E-PM) and baseline postural stability (eyes-open condition; COP E_0_) across the two tasks (Detection and Inhibition). Results indicated a significant main effect of cognitive complexity in both tasks (Detection: *F*(3, 95) = 5.866, *p* < 0.001, η_p_^2^ = 0.156; Inhibition: *F*(3, 95) = 6.629, *p* < 0.001, η_p_^2^ = 0.173), with COP being higher in all complexity conditions compared to the eyes-open (E_0_) baseline (all *p*s < 0.001). E_0_ represents the baseline condition without additional cognitive load, allowing effect sizes to reflect task-induced changes rather than absolute group differences. Thus, task manipulation resulted in a significantly higher COP compared to the condition without performing any additional task (i.e., E_0_), with Cohen’s *d* ranging between 1.01 (Condition E_0_ vs. E-PM/Inhibition task) and 1.90 (Condition E_0_ vs. I-PM/Inhibition task).

Results of the repeated-measures ANCOVA revealed a main effect of age group, *F*(1, 93) = 27.111, *p* < 0.001, η_p_^2^ = 0.226. As expected, younger adults showed a significantly lower CBCI than older adults (0.48 vs. 1.11). The task type x age group interaction was also significant (*F*(1, 93) = 13.601, *p* < 0.001, η_p_^2^ = 0.128), indicating lower CBCIs for the Detection task relative to the Inhibition task, with a more pronounced difference among older adults (0.84 vs. 0.44; Cohen’s *d* = 1.33). Moreover, post-hoc comparisons revealed that younger adults consistently had lower CBCIs across tasks compared to older adults (all *p*s < 0.001), with differences being more pronounced for the Inhibition task (0.83 vs. 0.43; Cohen’s *d* = 1.15).

A significant interaction between the level of complexity and age group was observed (*F*(2, 92) = 4.002, *p* < 0.05, η_p_^2^ = 0.080), revealing that younger adults had significantly lower CBCIs than older adults across all levels of cognitive complexity (all *p*s < 0.001). The largest difference was observed between E-PM compared to the Simple (0.84 vs. 0.52; Cohen’s *d* = 1.04) and the I-PM conditions (0.84 vs. 0.53; Cohen’s *d* = 1.04). Across age groups, CBCIs were consistently higher in the E-PM condition compared to the two other levels of complexity (all *p*s < 0.02), while the latter did not differ from each other (*p* = 0.85 for younger adults, *p* > 0.99 for older adults).

Additionally, a significant three-way interaction between age group, cognitive complexity, and task type revealed to be significant, *F*(2, 92) = 7.774, *p* < 0.001, η_p_^2^ = 0.145. Post-hoc comparisons revealed that, regardless of age or complexity, CBCIs were lower in the Detection task compared to the Inhibition task, with the most pronounced differences among older adults in the E-PM condition (all Cohen’s *d*s > 1.2). Moreover, post-hoc comparisons showed that, whatever the task and the level of cognitive complexity, younger adults displayed significantly lower CBCIs than older adults (*p* < 0.001), with age differences being more pronounced for the Inhibition task in the E-PM condition relative to all other comparisons (all Cohen’s *d*s > 1.02).

**Fig. 3:**
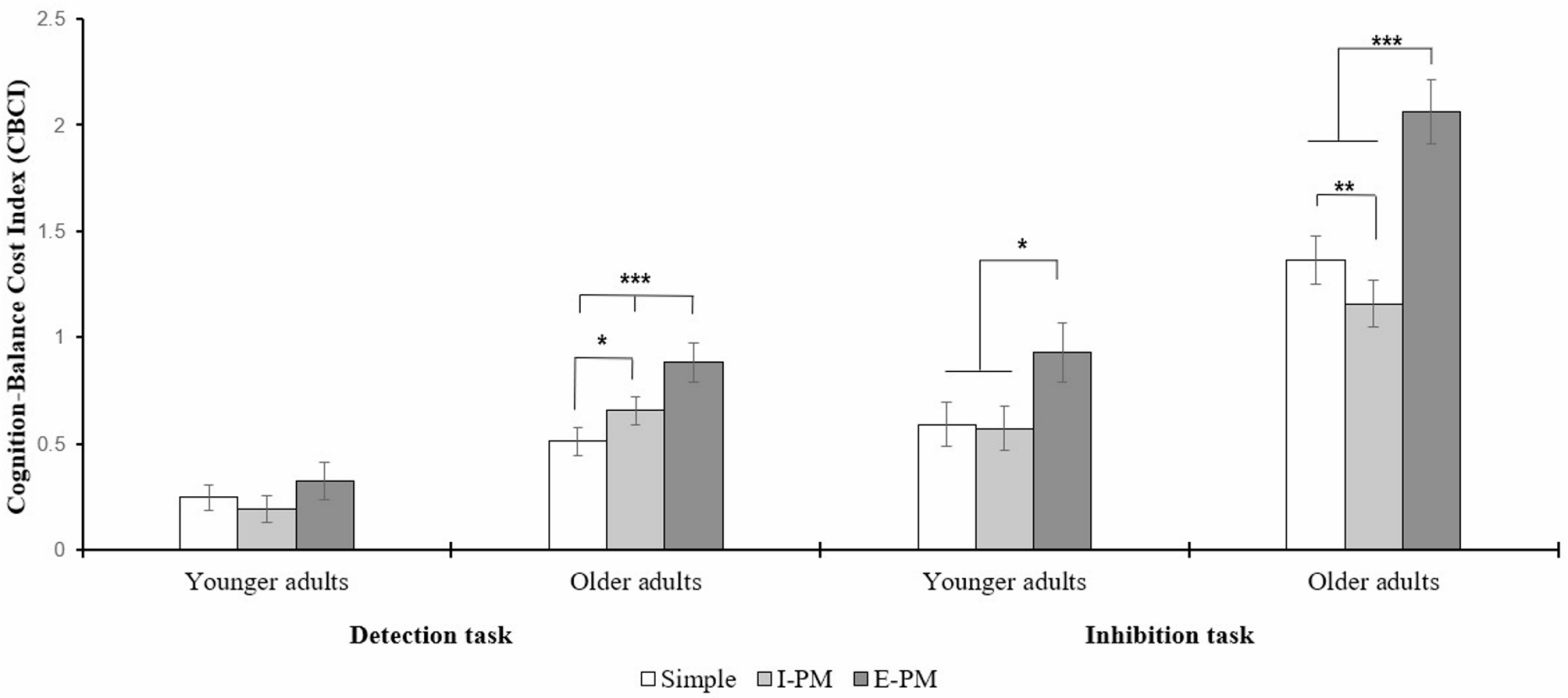
Differences in the cognition-balance cost index depending on task load and cognitive complexity in younger and older adults. Simple = simple task (i.e., without prospective memory task instruction); I-PM = intended prospective memory (i.e., secondary task instructed but without execution; deactivated intentions); E-PM = executed prospective memory (i.e., secondary task instructed with actual execution). Darker scaling indicates an increase in cognitive complexity. Significant post-hoc comparisons are marked by an asterisk. *p < 0.05. **p < 0.01. ***p < 0.001.

As shown in Fig. 3, among younger adults, the E-PM condition in the Inhibition task yielded significantly higher CBCIs than both the Simple and the I-PM condition. For older adults, in the Detection task, E-PM showed significantly higher CBCIs than I-PM, which in turn was higher than the Simple condition. However, in the Inhibition task, this hierarchy was reversed: the E-PM level showed the highest CBCIs, followed by the Simple, then the I-PM condition. This pattern suggests an inversion in the hierarchy of cognitive complexity under increased task difficulty. To disentangle whether this inversion is driven by reaction times or by COP, two complementary repeated-measures ANCOVA were performed on reaction times and COP, respectively. The results of these additional analyses revealed that, on the one hand, older adults were significantly slower in the I-PM condition compared to the simple condition. On the other hand, older adults showed a higher COP in the Inhibition task compared to the Detection task.

## Discussion

Walking while talking may be challenging for shared resources between cognitive and postural control. However, it is unknown whether merely keeping an intention in mind, even without executing it, represents cognitive load that can be detected through postural fluctuations. The present study investigated whether postural stability, as measured by center of pressure (COP), can serve as a task-external indicator of cognitive load induced by maintaining a PM intention. In this context, we propose the Cognition-Balance Cost Index (CBCI) as a proof-of-concept measure to detect and quantify potential trade-offs in postural stability through additional cognitive load. The present findings demonstrate that COP reveals additional costs associated with maintaining and executing intentions, particularly in older adults, supporting the idea that cognitive and postural control share common resources.

Consistent with theoretical frameworks emphasizing the cognitive demands of PM^14,16^, we observed that older adults exhibited higher CBCIs across all task conditions compared to younger adults. This finding aligns with prior research suggesting that age-related declines in attentional and executive resources increase the costs of maintaining an intention^20,21^. The most pronounced effects emerged when intentions were to be executed (E-PM condition), where the need to act on the intention likely amplified cognitive demands, resulting in increased postural instability. For younger adults, no significant differences were observed between the simple and the I-PM conditions, indicating that maintaining an intention did not impose a significant measurable cost in postural stability. This finding aligns with theories on cognitive development and previous evidence suggesting that younger adults – who are functioning well from a cognitive perspective – efficiently allocate cognitive resources without the need to sacrifice postural control^48,49,62–64^. However, the execution of an intention (E-PM) led to an increase in COP, suggesting that while *maintaining* an intention does not tax stability in this group – or at least not enough to be detected with the present index or the precision of the present material –, *executing* an intention introduces a transient cognitive load.

## Hierarchy of cognitive load and reserved effects in older adults

Another key finding concerns the hierarchy of cognitive complexity effects in older adults. In the Detection task, the CBCI increased significantly from the Simple condition to I-PM and further to E-PM, suggesting that maintaining an intention in mind implies a cognitive load, which is intermediate to the load induced by finally executing the intention. In the Inhibition task, however, participants showed a counterintuitive pattern: precisely, the I-PM condition resulted in lower CBCIs than the Simple condition. This suggests that maintaining an intention in the Inhibition task does not linearly increase cognitive load but may instead trigger a compensatory mechanism where attention is redirected to postural control^35,45,62,65^.

As the CBCI expresses the ratio between processing speed and posture, two concurrent reasons for a lower CBCI in the I-PM condition are plausible: On the one hand, the decrease in CBCI may be explained by a decrease in mean reaction time combined with a constant or greater postural instability (i.e., higher COP). This would imply, however, that older adults are faster in the I-PM condition compared to the Simple condition, which seems incongruous given the additional cognitive complexity. On the other hand, the decrease in CBCI could be due to an increase in postural instability without any change in response speed, meaning that older adults preferred cognitive performance over postural stability. To decide between these two alternative explanations, complementary analyses were conducted which revealed that older adults were significantly slower in the I-PM condition compared to the Simple condition, while also exhibiting increased postural instability in the Inhibition task.

Given that both mean reaction time and COP follow a parallel trajectory in the Detection task, the results observed in the Inhibition task (i.e., higher cognitive task load) allow to disentangle whether changes in CBCI are driven by reaction time or postural instability. The present findings indicate that the observed decrease in the index may be attributed primarily to an increase in postural instability rather than a disproportional improvement in cognitive efficiency. In line with previous research showing that individuals prioritize (maintaining) cognitive resources at the expense of postural accuracy in dual-task scenarios^66–68^, the present findings highlight the importance of considering postural control in aging populations, particularly when cognitive demands exceed available resources.

## Implications for aging and fall risk

The present results provide evidence that maintaining an intention in cognitive demanding tasks can impose hidden costs in older adults, which manifest in increased postural instability. This aligns with prior research showing that resource allocation shifts toward postural control in aging when cognitive resources are strained^41,46^. The findings have practical implications for understanding fall risk, as they suggest that cognitive load may amplify postural instability even in healthy older adults.

While prior research had predominantly examined cognitive load effects in dual-task paradigms^40,42^, the present study extends this research by demonstrating that cognitive demands related to intention maintenance alone can impact postural control. This emphasizes that fall risk in aging populations is not solely a matter of diminished equilibrium but involves a complex interaction between cognitive and postural resource allocation. Consequently, interventions aiming at reinforcing cognitive resources may help mitigate cascading effects of experiencing falls on subsequent cognitive decline in later life^69^.

From an applied perspective, explicit dual‑task trainings that combine balance exercises with PM tasks could be embedded into rehabilitation, with systematic progression along postural difficulty and intention load while using CBCI to identify an optimal dose and verify that cognitive gains are not purchased at the expense of postural stability. Also, virtual‑reality modules can recreate naturalistic environments (e.g., shopping, cooking, street crossing) to train intention maintenance and execution under controlled, increasingly complex sensory contexts while monitoring COP and CBCI in real time. In this context, compensatory strategies could be trained to manage multiple intentions safely: externalize cues, serialize intentions at balance‑critical moments, and apply stability‑first scripts (stabilize, then scan/execute), alongside environmental simplification in known high‑risk situations such as transitions, narrow base, or in poor lighting conditions. The low-cost nature of the force platforms used here enables broad deployment in clinics and community settings. Extending the application to wearables and smartphones-based technology allows continuous, real‑world monitoring of cognitive‑motor costs, which may ultimately serve AI‑assisted pattern detection to adapt reminder timing away from instability windows. In sum, CBCI can be incorporated into multifactorial fall‑risk assessments as an early marker of cognitive–postural resource competition, even when traditional measures are within normal limits.

## Limitations and future directions

A few limitations of the present study need to be acknowledged. First, we lacked a baseline PM condition in a standing-only context (i.e., without the imbalance platform). While we ensured that the eyes-open condition consistently resulted in lower COP values than the task conditions, a fully isolated PM condition would have strengthened the interpretation of our findings. Similarly, we did not include a sitting condition to establish a baseline for mean reaction time, although the index approach mitigates potential biases by allowing a parallel examination of both COP and reaction time effects. While these additional manipulations could have provided further reference points, they have already been widely examined in previous research. Given the additional participant burden, we focused on extending research gaps by prioritizing postural stability as a novel indicator of cognitive load in PM. Future studies may consider integrating these additional reference conditions into a fully comprehensive study protocol. In a similar direction, we acknowledge that the CBCI, as introduced here, represents a proof-of-concept measure. Full validation, including convergent validity with established dual-task cost measures and robustness under repeated testing within same conditions, particularly in patient populations, remains an important avenue for future research.

Second, the present cognitive task did not include a time-based PM condition and focused only on the intentional load associated with focal event-based PM cues. Specifically, participants had to react to a salient focal cue (i.e., red-colored arrows), which likely trigger intention retrieval through bottom-up processing. However, this task manipulation imposes a relatively low cognitive load compared to non-focal or time-based cues, which in turn demand more top-down processing and thus higher attentional/cognitive resources. The fact that COP measures were able to detect cognitive load even in this low-demanding condition highlights the sensitivity of postural imbalance as a task-external indicator of cognitive costs.

Last, the present sample consisted of healthy younger and older adults, limiting the generalizability of findings to populations at greater risk of falls or those with vestibular impairments. Future studies should investigate whether individuals with increased fall risk exhibit greater COP fluctuations under PM load. Furthermore, exploring intervention strategies, such as cognitive training or balance exercises, may provide insights into reducing the negative effects of cognitive load on postural stability in aging populations.

## Conclusion

Walking while talking (i.e., actively engaging in concurrent tasks) has been shown repeatedly to affect gait and postural control, but the effect of merely keeping a future intention in mind on postural resources has not been investigated. This study highlights postural stability, measured as COP, as a novel task-external indicator of cognitive load induced by maintaining an intention. By introducing the Cognition-Balance Cost Index (CBCI), which integrates reaction time and postural stability into a unified, proportional measure, this work provides a new, valuable approach to quantify cognitive-motor trade-offs beyond traditional behavioral outcomes. Our findings reveal that cognitive and postural resources are intertwined, particularly in older adults, where the cognitive demands of PM intentions can lead to increased postural instability. Notably, under lower task load, older individuals typically retain sufficient resource capacity to perform adequately. However, when faced with more demanding contexts – such as higher-order PM tasks – their cognitive system approaches its limits, resulting in heightened fluctuations in postural control. This pattern underscores that maintaining an intention induces a measurable cost in older adults. By bridging cognitive and motor research, these findings highlight the importance of considering postural stability in studies of cognitive aging. Future research needs to further explore how cognitive-postural interactions influence daily functioning and fall risk in older populations.

## Data Availability

The dataset generated during the current study and the annotated analytical scripts used are available on the Open Science Framework (OSF), https://osf.io/7trn5 .
